# Real world effectiveness of subcutaneous semaglutide in type 2 diabetes: A retrospective, cohort study (Sema-MiDiab01)

**DOI:** 10.3389/fendo.2022.1099451

**Published:** 2023-01-18

**Authors:** Cesare C. Berra, Maria Chiara Rossi, Marco Mirani, Daniela Ceccarelli Ceccarelli, Cristina Romano, Lorenza Sassi, Elena Peretti, Giuseppe Favacchio, Ida Pastore, Laura Folini, Giusi Graziano, Maria Elena Lunati, Sebastiano Bruno Solerte, Paolo Fiorina

**Affiliations:** ^1^ Department of Endocrine and Metabolic Diseases, I.R.C.C.S. MultiMedica – Sesto San Giovanni, Milan, Italy; ^2^ CORESEARCH – Center for Outcomes Research and Clinical Epidemiology, Pescara, Italy; ^3^ Department of Internal Medicine, I.R.C.C.S Humanitas Research Hospital – Rozzano, Milan, Italy; ^4^ Department of Internal Medicine, UOC Geriatrics and Diabetology, University of Pavia, Pavia, Italy; ^5^ Diabetology, Azienda Ospedaliera ASST Sette Laghi - Osp. di Circolo, Varese, Italy; ^6^ Division of Endocrinology, ASST Fatebenefratelli-Sacco, Milan, Italy; ^7^ International Center for T1D, Pediatric Clinical Research Center Romeo ed Enrica Invernizzi, DIBIC, Università di Milano, Milan, Italy

**Keywords:** type 2 diabetes, semaglutide, effectiveness, HbA1c, cardiovascular risk factors, beta-cell function, insulin resistance

## Abstract

**Introduction:**

Aim of the present study was to evaluate the real-world impact of once-weekly (OW) subcutaneous semaglutide on different end-points indicative of metabolic control, cardiovascular risk factors, and beta-cell function in type 2 diabetes (T2D).

**Methods:**

This was a retrospective, observational study conducted in 5 diabetes clinics in Italy. Changes in HbA1c, fasting blood glucose (FBG), body weight, blood pressure, lipid profile, renal function, and beta-cell function (HOMA-B) during 12 months were evaluated.

**Results:**

Overall, 594 patients (97% GLP-1RA naïve) were identified (mean age 63.9 ± 9.5 years, 58.7% men, diabetes duration 11.4 ± 8.0 years). After 6 months of treatment with OW semaglutide, HbA1c levels were reduced by 0.90%, FBG by 26 mg/dl, and body weight by 3.43 kg. Systolic blood pressure, total and LDL-cholesterol significantly improved. Benefits were sustained at 12 months. Renal safety was documented. HOMA-B increased from 40.2% to 57.8% after 6 months (p<0.0001).

**Discussion:**

The study highlighted benefits of semaglutide on metabolic control, multiple CV risk factors, and renal safety in the real-world. Semaglutide seems to be an advisable option for preservation of β-cell function and early evidence suggests it might have a role in modifying insulin resistance (HOMA-IR), the pathogenetic basis of prediabetes and T2D.

## Introduction

In spite of advances occurred in type 2 diabetes (T2D) therapy, achieving glycated haemoglobin (HbA1c) targets still represents a challenge for many patients (1). Multiple beneficial effects of glucagon-like peptide 1 receptor agonists (GLP1-RAs) in T2D care have been recognized by the most recent national and international guidelines (2, 3).

GLP-1 is an intestinal hormone which stimulates insulin secretion and inhibits pancreas glucagon secretion at carbohydrates intake (4). Due to its glucose-dependent action, GLP-1 secretion does not cause hypoglycaemia. The therapeutic class of GLP-1RAs mimics the effect of endogenous GLP-1, while having an increased half-life and a delayed subcutaneous absorption (5). In addition, during hypoglycaemia, GLP-1RAs can reduce secretion of insulin without hindering that of glucagon (6). Furthermore, GLP1-1RAs reduce appetite and preference for high-fat foods, with a positive effect on weight loss and body composition (7–9). GLP-1 receptors are also expressed in the heart, vascular system, immune system, and kidneys (4). In clinical studies, GLP-1RAs exerted a positive effect on glycated hemoglobin (HbA1c), body weight, and cardiovascular risk factors (plasma lipids, systolic blood pressure and inflammation) (10, 11). Their use in T2D is associated with lower risk of cardiovascular events (9).

The most recent (July 2021) Italian guidelines for the management and treatment of T2D recommend metformin, sodium-glucose co-transporter-2 inhibitors (SLGT-2is) or glucagon-like peptide-1 receptor agonists (GLP-1RAs) as first-line treatment options in patients with T2D and previous CV events (without heart failure) (12, 13). However, in Italy still low proportions of patients are treated with SGLT-2is or GLP-1RAs (14).

Semaglutide is the most recent available GLP-1RA; it has a sequence homology of 94% compared to human GLP-1 and is the first analogue available in both injectable and oral formulation (15). Semaglutide solution for injection in pre-filled pen has a prolonged half-life (165 hours), allowing for once-a-week (OW) administration; it was approved by the US Food and Drug Administration in December 2017 (16) and by the European Medicines Agency in February 2018 (17).

OW semaglutide is indicated in subjects with uncontrolled type 2 diabetes as a monotherapy when the use of metformin is contraindicated, or as an add-on therapy with other glucose-lowering drugs, including insulin (18, 19); furthermore, based on the most recent guidelines, it can be considered for use as a first-line therapy in T2D patients with previous CV event (2, 20).

The efficacy of OW subcutaneous (s.c.) semaglutide was evaluated in over 7000 patients in six global phase IIIa trials and in nearly 3000 patients in four phase IIIb trials (SUSTAIN program) (21). OW semaglutide is currently recognized as the most powerful molecule within the GLP-1RAs class (22). In fact, it has a great efficacy in the improvement of glycaemic control (up to 1.8% of HbA1c reduction in the different studies) and reduction in body weight (up to -6.5 Kg in the different studies) in T2D patients.

In the SUSTAIN-6 study, involving patients with T2D with high cardiovascular risk, a 26% statistically significant risk reduction of major cardiovascular events was documented for OW semaglutide vs. placebo (23).

However, current evidence on OW semaglutide mainly derives from controlled clinical studies, conducted on selected populations for a limited period of observation and treatment. The strict inclusion/exclusion criteria applied in randomized controlled trials (RCTs) often result in a patient population that is not fully representative of the range of patients treated in routine clinical practice. As known, real-world studies allow to assess whether the results of experimental studies can be reproduced in broader samples of patients managed under routine clinical practice (24). Therefore, the aim of the present study was to assess effectiveness and safety of OW semaglutide when used in T2D patients managed by a network of diabetes clinics.

## Materials and methods

This was a multicenter, observational, retrospective study, aiming to investigate the use and the impact of OW semaglutide after 6 and 12 months of treatment. The study involved 5 centres located in the Lombardia region (Italy).

Inclusion criteria were: diagnosis of T2D according to ADA definition for at least 3 months, men or women aged > 18 years, stable antihyperglycemic therapy for at least 3 months with oral hypoglycaemic agents (OHA) and/or insulin, prescription of OW semaglutide according to routine clinical practice, and signed informed consent. Semaglutide in T2D is reimbursed in Italy by the national healthcare system.

Exclusion criteria were: any patient condition precluding adequate understanding of informed consent, other diabetes types, and previous or current participation of patients in interventional clinical studies.

Patients were treated with OW s.c. semaglutide in prefilled pen injector, according to routine clinical practice. The treating physician determined the maintenance dose of semaglutide and any subsequent changes to this dose.

Data were retrospectively collected from the same electronic medical record system adopted in the different participating centers (Smart digital clinic) for the routine management of patients. No additional diagnostic or monitoring procedures were implemented on patients outside normal clinical practice. Baseline was represented by the visit where the patient received the first prescription of semaglutide (T0); follow-up visits were those performed after 3 (T3), 6 (T6) and 12 (T12) months from baseline, according to Italian Standards of Diabetes Care.

The following data were collected at baseline: gender, age, ethnicity, T2D duration, HbA1c, fasting blood glucose (FBG), body mass index (BMI), waist circumference, blood pressure (BP), doses of OW semaglutide, total cholesterol, high-density lipoprotein (HDL) cholesterol, triglycerides, calculated low-density lipoprotein (LDL) cholesterol, serum creatinine, estimated glomerular filtration rate (e-GFR), albumin-creatinine ratio (ACR), transaminases (Alanine transaminase - ALT, aspartate aminotransferase - AST), diagnosis of liver steatosis, heart rate, diabetes complications (diabetic retinopathy, ischemic heart disease, heart failure, cerebral vascular disease, ischemic transient attacks or stroke, peripheral vascular disease, supra-aortic trunks vasculopathy), glucose-lowering, antihypertensive and lipid-lowering therapy, fasting insulinemia, homeostatic model assessment for insulin resistance (HOMA-IR) and for β-cell function (HOMA-B).

At follow-up visits (at 3, 6 and 12 months) the following data were collected: HbA1c, FBG, weight, BMI, blood pressure, lipid profile, eGFR, ACR, HOMA-IR, HOMA-B, treatments, doses of OW semaglutide, hypoglycemia, and semaglutide discontinuation.

All data were anonymous.

Primary endpoint was the change in mean HbA1c levels from baseline to 6 months. Continuous secondary endpoints were: change in mean HbA1c levels from baseline to T3 and T12 months, change in mean FBG levels and weight/BMI from baseline to T3, T6, and T12 months, and changes from baseline to T6 and T12 in the following parameters: blood pressure, lipid profile, eGFR, ACR, and HOMA-B.

Categorical secondary endpoints were: percentage of patients with HbA1c ≤7%, HbA1c >8.0%, BMI ≥30 Kg/m^2^, BP ≤140/90mg/dl, LDLc <100mg/dl, ACR between 30 and 300 or ACR >300 mg/g, eGFR <60 ml/min*1.73 m^2^), HOMA-IR >2.5, patients with no, mild (54-70 mg/dl), moderate (<54 mg/dl), and severe (need of third- party assistance) hypoglycemia, patients discontinuing semaglutide and reasons for discontinuation.

### Statistical analysis

A minimum sample size of 100 subjects allowed to detect with a statistical power of 80% a minimum reduction of HbA1c of 0.6% at T6 (slightly lower than that obtained in RCTs but reflecting the greater variability of results deriving from a “real life” clinical experience, taking into account also of the variability of associated therapies), and assuming a baseline standard deviation of HbA1c of 2.0% (larger than that reported in RCTs due to the greater variability expected in an observational setting), and with a significance level (alpha error) of 0.05%.

Descriptive data were summarized as mean and standard deviation for continuous variables or proportion for categorical variables.

Changes in continuous study endpoints were assessed using mixed models for repeated measurements. Results are expressed as estimated mean or estimated mean difference from T0 with their 95% confidence interval (95% CI). Paired t-test derived from linear mixed models for repeated measurements were applied for pre-post within group comparisons.

Changes in categorical study endpoints were assessed through the chi-square test for trend.

Statistical significance was defined by a p-value was < 0.05.

## Results

Overall, 459 T2D patients with first prescription of semaglutide were seen in the 5 participating centres between February 2019 and January 2022. Among them, 14 patients (3.0%) were already treated with a GLP1-RAs, while the remainders were naïve to GLP-1 treatment. Baseline patient characteristics are reported in **Table 1**. Mean age was 63.9 ± 9.5 years, 62.7% of participants were male, mean diabetes duration was 11.4 ± 8 years. At semaglutide initiation (T0), average HbA1c was 7.8 ± 1.3% and average BMI was 32.5 ± 5.6 Kg/m^2^; all patients were treated with 1 or more OHAs, while 23.5% of patients were treated with basal insulin + OHAs. About half of subjects (52.4%) had diabetes complications and approximately 80% of them were treated with antihypertensive and lipid lowering drugs.

**Table 1:** Baseline patients’ characteristics.

	% or mean ± std
**N**	459
**Men (%)**	62.7
**Age (years)**	63.9 ± 9.5
**Not Caucasian ethnicity (%)**	0.9
**T2D duration (years)**	11.4 ± 8.0
**Smokers-ex smokers (%)**	45.9
**HbA1c (%)**	7.8 ± 1.3
**FBG (mg/dl)**	159.8 ± 49.7
**BMI (Kg/m^2^)**	32.5 ± 5.6
**Waist circumference (cm)**	113.0 ± 12.0
**SBP (mmHg)**	132.2 ± 14.2
**DBP (mmHg)**	79.7 ± 8.6
**Total cholesterol (mg/dl)**	167.4 ± 39.8
**LDL- cholesterol (mg/dl)**	91.2 ± 38.5
**HDL- cholesterol (mg/dl)**	45.1 ± 11.0
**Triglycerides (mg/dl)**	168.5 ± 115.6
**Creatinine (mg/dl)**	1.0 ± 0.4
eGFR (CKD-Epi formula) (ml/min*1.73m^2^)	77.6 ± 20.9
**ACR (mg/mmol)**	85.4 ± 356.7
**ALT (iu/l)**	36.3 ± 24.0
**AST (iu/l)**	34.7 ± 77.1
**Steatosis (%)**	73.1
**Heart rate (bpm)**	76.0 ± 11.9
**Diabetes complications (%):**	52.4
** Ischemic heart disease**	24.1
** Heart failure**	8.3
** Cerebral vasculopathy**	6.2
** Peripheral vasculopathy**	10.9
** Supra-aortic trunks vasculopathy**	43.4
** Retinopathy (%)**	12.3
Glucose-lowering drugs before starting semaglutide (%):	
** Metformin**	81.7
** Pioglitazone**	9.4
** Sulphonylureas**	13.3
** DPPIVi**	13.3
** SGLT2i**	16.5
** GLP1-RA**	3.0
** Basal insulin**	23.5
** Short-acting insulin**	6.7
**Antihypertensive treatment (%)**	79.2
**Lipid-lowering treatment (%)**	77.5
**Fasting insulinemia (microU/ml)**	13.4 ± 10.6
**HOMA-IR**	5.0 ± 3.6
**HOMA-B (%)**	40.3 ± 24.0

HbA1c, Glycated haemoglobin; FBG, Fasting blood glucose; BMI, body mass index; SBP, Systolic blood pressure; DBP, Diastolic blood pressure; HDL, High-density lipoprotein; LDL, Low-density lipoprotein; eGFR, estimated glomerula filtration rate; ACR, albumin creatinine ratio; ALT, Alanine transaminase; AST, aspartate aminotransferase; DPP-IVi, Inhibitors of dipeptidyl peptidase 4; SGLT2i, sodium-glucose co-transporter-2 inhibitors; GLP1-RA, glucagon-like peptide 1 receptor agonists; HOMA-IR, homeostatic model assessment for insulin resistance; HOMA-B, homeostatic model assessment for β-cell function.

**Figure 1:**
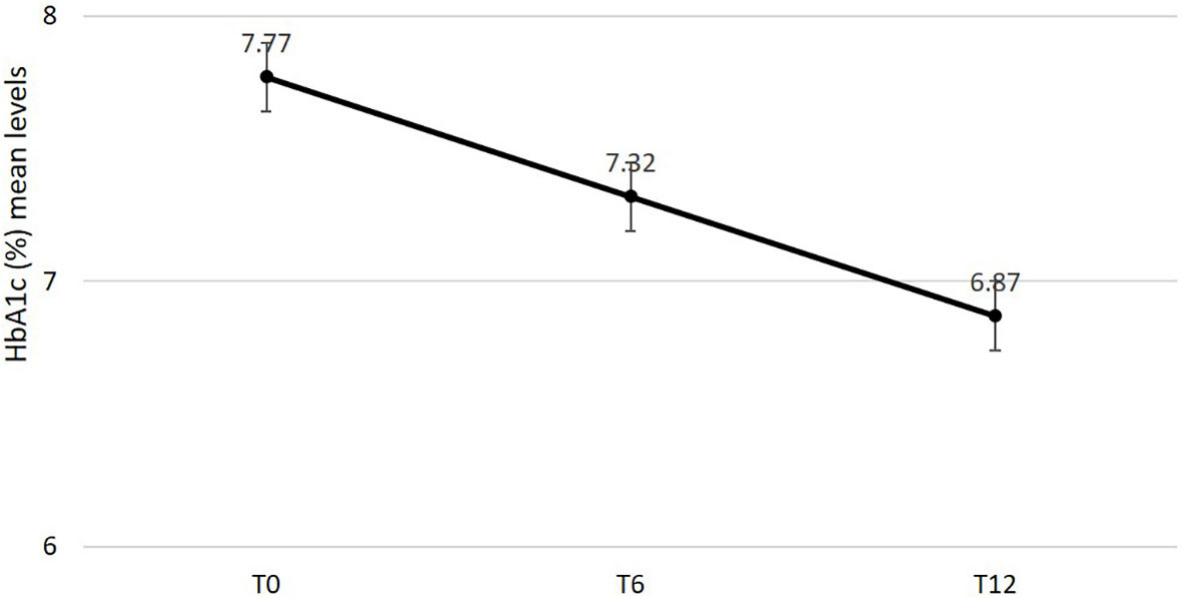
Changes in estimated mean levels of HbA1c after 6 and 12 months from semaglutide initiation.

Effectiveness data are reported in **Table 2**. After 6 and 12 months mean changes in HbA1c (**Figure 1**), FBG and body weight were statistically significant and clinically relevant. Specifically, HbA1c decreased by -0.9% (95% C.I. -1.04; -0.76, *p* < 0.0001) after 6 months and the reduction was sustained after 12 months (-0.96%; 95% C.I. -1.09; -0.82, *p* < 0.0001); FBG decreased by -26.24mg/dl (95% C.I. -32.25; -20.23, *p* < 0.0001) after 6 months and the reduction was sustained after 12 months (-25.76 mg/dl; 95% C.I. -31.57; -19.94, *p* < 0.0001); body weight was reduced by -3.43 kg (95% C.I. -4.51; -2.34, *p* < 0.0001) after 6 months and benefit was substantially maintained after 12 months (-3.68 kg; 95% C.I.-4.93; -2.44, *p* < 0.0001). A statistically significant reduction in systolic but not diastolic blood pressure mean levels was documented after 6 and 12 months. No changes were documented in renal function. Total cholesterol and LDL cholesterol levels significantly improved over 12 months. A statistically significant and clinically relevant improvement in HOMA B was documented at 6 months (+17.53; 95% C.I. 14.21; 20.85, *p* < 0.0001). No data on HOMA B at 12 months were available.

**Table 2:** Changes in estimated mean levels of continuous clinical endpoints over time.

Change in	Visit	Estimated mean and 95% CI	Estimated mean difference from T0 and 95% CI	Within group p-value*

HbA1c (%)	T0	7.77		
		(7.65;7.89)		
	T3	7.32	-0.45	**<0.0001**
		(7.11;7.52)	(-0.64;-0.26)	
	T6	6.87	-0.9	**<0.0001**
		(6.74;7.01)	(-1.04;-0.76)	
	T12	6.82	-0.96	**<0.0001**
		(6.7;6.94)	(-1.09;-0.82)	
FBG (mg/dl)	T0	159.77		
		(155.07;164.47)		
	T3	138.85	-20.92	**<0.0001**
		(132.56;145.14)	(-28.06;-13.78)	
	T6	133.53	-26.24	**<0.0001**
		(128.82;138.24)	(-32.25;-20.23)	
	T12	134.01	-25.76	**<0.0001**
		(129.3;138.73)	(-31.57;-19.94)	
Body weight (Kg)	T0	92.1		
		(90.41;93.78)		
	T3	91.11	-0.99	0.22
		(89.09;93.13)	(-2.57;0.59)	
	T6	88.67	-3.43	**<0.0001**
		(86.76;90.59)	(-4.51;-2.34)	
	T12	88.41	-3.68	**<0.0001**
		(86.61;90.22)	(-4.93;-2.44)	
BMI (Kg/m^2^)	T0	32.62		
		(32;33.25)		
	T3	32.1	-0.53	**<0.0001**
		(31.45;32.74)	(-0.69;-0.36)	
	T6	31.64	-0.99	**<0.0001**
		(31;32.27)	(-1.19;-0.79)	
	T12	31.25	-1.38	**<0.0001**
		(30.61;31.88)	(-1.62;-1.13)	
SBP (mmHg)	T0	132.41		
		(130.86;133.96)		
	T6	130.09	-2.32	**0.03**
		(127.92;132.26)	(-4.43;-0.2)	
	T12	130.18	-2.22	0.05
		(128.04;132.32)	(-4.49;0.04)	
DBP (mmHg)	T0	79.79		
		(78.86;80.72)		
	T6	78.53	-1.27	0.06
		(77.31;79.75)	(-2.59;0.06)	
	T12	79.59	-0.2	0.78
		(78.37;80.82)	(-1.56;1.17)	
Total cholesterol (mg/dl)	T0	167.07		
		(162.87;171.28)		
	T6	155.61	-11.47	**<0.0001**
		(149.86;161.35)	(-17.19;-5.75)	
	T12	157.99	-9.09	**0.0004**
		(152.85;163.12)	(-14.08;-4.09)	
LDL cholesterol (mg/dl)	T0	91.13		
		(87.05;95.21)		
	T6	81.88	-9.25	**0.0002**
		(77.03;86.73)	(-14.05;-4.45)	
	T12	84.69	-6.44	**0.01**
		(80.14;89.23)	(-11.37;-1.51)	
HDL cholesterol (mg/dl)	T0	45.33		
		(44.18;46.49)		
	T6	44.99	-0.34	0.57
		(43.49;46.49)	(-1.52;0.83)	
	T12	46.13	0.79	0.16
		(44.79;47.46)	(-0.31;1.89)	
Triglycerides (mg/dl)	T0	166.51		
		(154.3;178.71)		
	T6	153.01	-13.49	0.07
		(140.62;165.4)	(-27.9;0.91)	
	T12	147.18	-19.33	**0.001**
		(135.33;159.03)	(-31.1;-7.56)	
eGFR (ml/min*1.73m^2^)	T0	77.57		
		(75.52;79.61)		
	T6	77.1	-0.47	0.44
		(74.98;79.22)	(-1.65;0.72)	
	T12	77.92	0.36	0.55
		(75.77;80.07)	(-0.81;1.52)	
ACR (mg/g)	T0	84.39		
		(27.31;141.47)		
	T6	34.26	-50.13	0.18
		(-15.48;84)	(-123.76;23.49)	
	T12	49.29	-35.09	0.33
		(2.9;95.69)	(-105.91;35.72)	
HOMA-B (%)	T0	40.28		
		(32.05;48.52)		
	T6	57.81	17.53	**<0.0001**
		(48.96;66.66)	(14.21;20.85)	

HbA1c, Glycated haemoglobin; FBG, Fasting blood glucose; BMI, body mass index; SBP, Systolic blood pressure; DBP, Diastolic blood pressure; HDL, High-density lipoprotein; LDL, Low-density lipoprotein; eGFR, estimated glomerula filtration rate; ACR, albumin creatinine ratio; HOMA-B, homeostatic model assessment for β-cell function; 95% CI, 95% confidence intervals.

*Paired t-test derived from linear mixed models for repeated measurements. Values in bold are statistically significant

As for categorical endpoints (**Table 3**), HbA1c levels <7% were reached by 65.2% of patients after 12 months of treatment and just 10.8% of them showed HbA1c levels >8%. A significant reduction in the proportion of patients with BMI ≥ 30 kg/m^2^ was documented. Finally, an improvement in HOMA-IR was registered after 6 months of treatment, with a reduction in the percentage of patients with HOMA-IR >2.5, although the statistical significance was not reached; no data on HOMA-IR at 12 months were available.

**Table 3:** Changes in categorical endpoints.

Endpoint	T0	T6	T12	p-value*
HbA1c ≤7%	30.4	68.9	65.2	**<0.0001**
HbA1c >8%	33.1	15	10.8	**<0.0001**
BMI ≥ 30 Kg/m^2^	65.2	62.4	49.4	**<0.0001**
BP ≤140/90 mg/dl	83.0	85.8	86.7	0.65
LDL-c <100 mg/dl	65.7	72.0	74.5	0.10
ACR:				1.00
<30 mg/g	74.8	81.5	76.1	
30-300 mg/g	20.3	14.8	20.5	
>300 mg/g	4.9	3.7	3.4	
eGFR <60 mg/dl	20.5	16.3	18.8	0.12
HOMA-IR >2.5	75.5	50.0	–	0.59
Hypoglycemia:				0.70
None	–	99.5	98.3	
Mild (54-70 mg/dl)	–	0.5	1.1	
Moderate (<54 mg/dl)	–	0	0	
Severe (need of third-party assistance)	–	0	0.6	

chi square for trend. Values in bold are statistically significant.

According to the summary of product characteristics, all patients were first prescribed with semaglutide 0.25 mg during the first month; semaglutide was then titrated up to 0.50 mg and in some cases up to 1.0 mg (based on patient needs). In this cohort, 15.7% patients were treated with 1.0 mg after 12 months of treatment.

Patients discontinuing semaglutide during 12 months were a small minority (N=4). Reasons for discontinuation were: elevation in lipase levels (1 patient at 6 months and 1 patient at 12 months), elevation in lipase and amylase levels (1 patient at 12 months), and intolerance (1 patient at 12 months).

Patients experiencing hypoglycemia were a small minority: an episode of mild (54-70 mg/dl) hypoglycemia was registered in 0.5% of patients at T6 and 1.1% at T12; at T12, 1 episode of severe hypoglycemia was reported.

No major changes in concomitant glucose-lowering, antihypertensive, and lipid-lowering therapies were recorded during 12 months.

## Discussion

The present study confirms the effectiveness and tolerability of OW semaglutide in a high CV risk real-world cohort of patients with uncontrolled T2DM. In this predominantly GLP1-RA naïve patients, starting OW semaglutide translated into marked benefits on HbA1c (about 1% decrease after 6 months, sustained at 12 months), and weight (-3.5 Kg). These results mirror those obtained in the SUSTAIN studies in patients treated with 0.5 mg, likewise most patients in this real-world study (16, 23, 25–31).

Furthermore, improvements in FBG, BMI, SBP, total and LDL cholesterol, and triglycerides were documented, in line with existing knowledge (4, 9, 20, 32–38). Renal parameters (eGFR and ACR) were unchanged after 12 months of treatment, confirming the renal safety of GLP1-RAs which were demonstrated to prevent the onset of macroalbuminuria and slow the decline of eGFR (39).

All these benefits were obtained without changing glucose-lowering, antihypertensive and lipid-lowering drugs.

Moreover, only 15.7% of patients in the study achieved a semaglutide dose of 1.0 mg at after 12 months. This picture could have been influenced by COVID-19 pandemic which was responsible for a reduction in clinic visits and highlights the need to help patients reach a 1.0 mg dose in the real-world settings when needed. However, it is important to note that relevant clinical benefits on metabolic parameters and CV risk factors have been achieved also with intermediate dosage. These results are consistent with those of other real-world studies based on cohorts followed in other settings, all documenting effectiveness and safety of OW semaglutide (32, 35, 40–47).

In addition, this study assessed changes in HOMA-IR and HOMA-B, seldom investigated in the other studies, suggesting improvements both in beta-cell function and insulin resistance. These results are in line with a recent network meta-analysis documenting that incretin-based therapies not only support an increase in HOMA-B, but also achieve a reduction in HOMA-IR in comparison with placebo; although GRADE scores indicate low to moderate quality for most of these comparisons, incretin-based therapies seem to be an advisable option for long-term treatment to preserve β-cell function (48).

In T2D patients eligible for GLP-1 therapy, HOMA-IR before starting the treatment could be an indicator of patients’ “phenotype” as defined by Ahlqvist et al. (49). HOMA-IR might also help to anticipate patient’s response to incretin-based therapies. There is limited current evidence to establish whether HOMA-IR amelioration with GLP-1 is an independent outcome or a secondary result from weight loss and body recomposition (50, 51).

It is known that T2D is a chronic degenerative disease characterized by high risk of complications and an increasing social, economic and health burden worldwide (52, 53); on the other hand, GLP-1 therapy is recognized as one of the key strategies to overcome clinical inertia in cardiorenal protection of T2DM patients (53, 54).

The study has strengths and limitations. Main strengths were the large sample size from different outpatient clinics (a multicentre cohort), the inclusion of many endpoints, some of them seldom investigated, and the follow-up exceeding 32 weeks (timeframe of RCTs). In particular, some of the endpoints we considered emphasize the relevance of non-glycaemic targets of treatment such as β-cell function and insulin resistance (HOMA-IR). The former is a marker of disease progression to the insulin – dependent phase of T2D, while insulin resistance has been demonstrated to be an independent risk factor for cardiovascular, neurodegenerative, and other diseases (55, 56). Another strength is the inclusion of a real-world population of patients with T2D that is different from that typically enrolled in RCTs. Patients with a wide range of baseline characteristics were included, such as those treated with different medications at baseline.

Among limitations, it should be acknowledged the lack of data on HOMA-B and HOMA-IR for a large proportion of patients and no information on glycaemic variability. Retrospectively, these limitations point out the importance of collecting data on beta-cell function/HOMA-B and insulin resistance/HOMA-IR at first patients’ evaluation, even in real world setting, in order to design a treatment pathway that goes beyond glycaemic targets and addresses the features of T2D in each single patient.

In conclusion, the study documented benefits of OW semaglutide on metabolic control, multiple CV risk factors, and renal safety in the real-world. Semaglutide seems to be an advisable option for preservation of β-cell function. Emerging evidence indicates that semaglutide could have disease-modifying actions on insulin resistance (HOMA-IR) and the early stage of prediabetes and T2D; nevertheless, further data are needed to establish whether this is an independent treatment outcome or a consequence of weight and fat loss.

## Data availability statement

The raw data supporting the conclusions of this article will be made available by the authors, without undue reservation.

## Ethics statement

The studies involving human participants were reviewed and approved by the master Ethics committee (Comitato Etico Milano Area 1 protocol number 2021/ST/90) and by Ethics Committees of all participating centers. The patients/participants provided their written informed consent to participate in this study.

## Author contributions

CB and PF contributed to the study conception and design. Data were collected by CR, MM, DC, CR, LS, EP, GF, IP, LF, ML, SS and PF. Data analysis was performed by MR and GG. The first draft of the manuscript was written by CB and MR and all authors commented on previous versions of the manuscript. All authors read and approved the final manuscript.
